# miR-592 acts as an oncogene and promotes medullary thyroid cancer tumorigenesis by targeting cyclin-dependent kinase 8

**DOI:** 10.3892/mmr.2021.12312

**Published:** 2021-07-22

**Authors:** Ting Liu, Jingjing Meng, Yu Zhang

Mol Med Rep 22: 3316-3326, 2020, DOI: 10.3892/mmr.2020.11392

Following the publication of this paper, the authors have realized that they overlooked indicating that Ting Liu and Jingjing Meng contributed equally to this work. Therefore, the affiliations for this paper should have been written as follows:

TING LIU^1*^, JINGJING MENG^2*^ and YU ZHANG^3^

Departments of ^1^Nuclear Medicine and ^2^Thyroid and Breast Surgery, The Affiliated Wuhan Central Hospital of Tongji Medical College, Huazhong University of Science and Technology, Wuhan, Hubei 430014; ^3^Department of Surgery II, Shanghai Municipal Hospital of Traditional Chinese Medicine, Shanghai University of Traditional Chinese Medicine, Shanghai 200071, P.R. China

*Contributed equally

Furthermore, the authors have also realized that they overlooked acknowledging a source that contributed towards the paper's funding in the Funding section of the declarations. Accordingly, the following information should have been included in the paper regarding the funding received:

## Notes

The authors confirm that there are no further errors in the study, and all the authors agree to this correction. The authors regret their oversight, and apologize for any inconvenience caused.

